# Das Hamburgische, Collegium Medicam, Und Der Artzliche Verein in Hamburg

**Published:** 1841-07-03

**Authors:** 


### BIBLIOGRAPHICAL NOTICE.

*Das Hamburgische, Collegium Medicam, und der Artzliche Verein in Hamburg.* Von Nicolaus Friedrich Schrader, Dr. Med. et Chir.

*The Hamburg Medical College, and Union of Physicians in Hamburg.* By Nicholas Frederic Schrader, Doctor of Medicine and Surgery.

This is a history of the Medical Society of Hamburg, with the names of members, and indications of the subjects of thesis of those who were formerly members. It was prepared on the celebration of the twenty-fifth anniversary of the revival of the society. To the members of the society, it must be highly interesting.

---

We have received, through the kindness of Dr. Oppenheim, another pamphlet, being the address of Dr. Siemer, President of this Society.

It seems that former attempts at founding a medical society failed, from want of unity among the members; this defect, Dr. Siemer states, is now remedied, and the 143 physicians of Hamburg, and 36 of Altona, live together on very friendly terms. Still, there, as elsewhere, "it is not possible that sometimes a misunderstanding should not arise. The elements are too heterogeneous to form a uniform whole. Veniam damus, petinius que vicissim."

The whole pamphlet gives us the impression that the relations of the physicians of Hamburg are of a very agreeable kind, and that, as the author states, they differ more about ideas than persons. The object of this society or union is to continue this friendly feeling; and although, on the one hand, the want of union depends upon the very nature of our art, which will probably never attain mathematical certainty, on the other this very diversity may be useful and agreeable; "but we must separate the person from the thing,"—that is, the scientific pursuit in which many different modes of culture are allowable.

The Society finally, on the recommendation of Dr. Siemer, elected a number of honorary and corresponding members; the distinction, we suppose, being that honorary members are supposed to be past work, and corresponding still active,—at least, such is the construction that our own position in the list entitles us to take. Five corresponding members have been named in the United States, — the Society selecting the representatives from the editorial corps, as among the most useful members of the profession, or, at least, among those who have the hard task of writing or compiling more matter than is always read. They are Dr. J. V. C. Smith, of Boston, and Drs. Hays, Dunglison, Biddle and Gerhard, of this city.

